# 高效液相色谱-四极杆-飞行时间质谱快速筛查鉴别食品中非法添加的62种中药材

**DOI:** 10.3724/SP.J.1123.2021.02031

**Published:** 2022-01-08

**Authors:** Wanqin WU, Feng JIANG, Xiaolong FAN, Ran CHEN, Xiaoling ZHU, Qi CAO, Zhengwei ZHU, Songsong ZHU, Huixia WANG

**Affiliations:** 湖北省食品质量安全监督检验研究院, 湖北省食品质量安全检测工程技术研究中心, 湖北 武汉 430075; Hubei Provincial Institute for Food Supervision and Test, Hubei Provincial Engineering and Technology Research Center for Food Quality and Safety Test Office, Wuhan 430075, China; 湖北省食品质量安全监督检验研究院, 湖北省食品质量安全检测工程技术研究中心, 湖北 武汉 430075; Hubei Provincial Institute for Food Supervision and Test, Hubei Provincial Engineering and Technology Research Center for Food Quality and Safety Test Office, Wuhan 430075, China; 湖北省食品质量安全监督检验研究院, 湖北省食品质量安全检测工程技术研究中心, 湖北 武汉 430075; Hubei Provincial Institute for Food Supervision and Test, Hubei Provincial Engineering and Technology Research Center for Food Quality and Safety Test Office, Wuhan 430075, China; 湖北省食品质量安全监督检验研究院, 湖北省食品质量安全检测工程技术研究中心, 湖北 武汉 430075; Hubei Provincial Institute for Food Supervision and Test, Hubei Provincial Engineering and Technology Research Center for Food Quality and Safety Test Office, Wuhan 430075, China; 湖北省食品质量安全监督检验研究院, 湖北省食品质量安全检测工程技术研究中心, 湖北 武汉 430075; Hubei Provincial Institute for Food Supervision and Test, Hubei Provincial Engineering and Technology Research Center for Food Quality and Safety Test Office, Wuhan 430075, China; 湖北省食品质量安全监督检验研究院, 湖北省食品质量安全检测工程技术研究中心, 湖北 武汉 430075; Hubei Provincial Institute for Food Supervision and Test, Hubei Provincial Engineering and Technology Research Center for Food Quality and Safety Test Office, Wuhan 430075, China; 湖北省食品质量安全监督检验研究院, 湖北省食品质量安全检测工程技术研究中心, 湖北 武汉 430075; Hubei Provincial Institute for Food Supervision and Test, Hubei Provincial Engineering and Technology Research Center for Food Quality and Safety Test Office, Wuhan 430075, China; 湖北省食品质量安全监督检验研究院, 湖北省食品质量安全检测工程技术研究中心, 湖北 武汉 430075; Hubei Provincial Institute for Food Supervision and Test, Hubei Provincial Engineering and Technology Research Center for Food Quality and Safety Test Office, Wuhan 430075, China; 湖北省食品质量安全监督检验研究院, 湖北省食品质量安全检测工程技术研究中心, 湖北 武汉 430075; Hubei Provincial Institute for Food Supervision and Test, Hubei Provincial Engineering and Technology Research Center for Food Quality and Safety Test Office, Wuhan 430075, China

**Keywords:** 高效液相色谱-四极杆-飞行时间质谱, 筛查鉴别, 非法添加, 中药材, 食品, high performance liquid chromatography-quadrupole-time of flight mass spectrometry (HPLC-Q-TOF/MS), screening and identification, illegally added, traditional Chinese medicine (TCM), food

## Abstract

建立了高效液相色谱-四极杆-飞行时间质谱快速筛查鉴别食品中非法添加的62种中药材的方法。依据卫生部关于进一步规范保健食品原料管理的通知(卫法监发[2002]51号)中可用于保健食品的物品名单,确定食品中可能非法添加的62种中药材原料清单,再从62种中药材中筛选其特征组分,获得不同中药材对应的特征组分清单。62种对照药材经甲醇超声提取后,于Thermo Accucore aQ色谱柱(150 mm×2.1 mm, 2.6 μm)上分离,在电喷雾正负离子扫描模式下,分别以0.1%(v/v)甲酸水溶液-乙腈和水-乙腈为流动相梯度洗脱,进行一级质谱和二级质谱全扫描检测,采用Library View软件建立不同中药材对应的特征组分的一级精确质量数据库和二级碎片质谱库。样品同法处理后上样分析,采用Peak View软件将样品高分辨数据与自建数据库中的质谱图、精确分子离子质量数、碎片离子质量数、保留时间等相关参数进行快速筛查鉴别分析。该工作通过建立“中药材-特征组分”对应清单,构建了食品中易非法添加的62种中药材中共388种特征组分的高分辨质谱库,每种中药材包括5~10种特征组分,通过对实际食品样品配制酒、代用茶及饮料进行筛查分析,1批次配制酒样品与淫羊藿中药材的7种特征组分匹配一致,推断该配制酒样品中加入了淫羊藿中药材。该法可实现无标准品情况下中药材的定性筛查,具有高通量、准确、简便、快捷的特点,解决了食品中非法添加中药材难以识别和确证的难题,可以实现食品中非法添加中药材的快速筛查鉴别分析。

## Abstract (en)

An analytical method based on high performance liquid chromatography coupled with quadrupole time-of-flight mass spectrometry (HPLC-Q-TOF/MS) was established for the rapid screening and identification of 62 kinds of illegally added traditional Chinese medicine (TCM) in food. According to the notice of the Ministry of Health of the People’s Republic of China on further regulating the management of raw materials of health food (Weifa Jianfa (2002) No. 51), the characteristic components of the 62 kinds of TCM were screened, and the corresponding characteristic component lists of different TCM were obtained. Methanolic extracts of the 62 kinds of standard medicinal materials were subjected to HPLC-Q-TOF-MS analysis. The filtrate was separated on a Thermo Accucore aQ column (150 mm×2.1 mm, 2.6 μm) using 0.1%(v/v) formic acid aqueous solution or water and acetonitrile as the mobile phases for gradient elution in the electrospray positive and negative ion scanning mode. All the data were determined on the full scan of primary mass spectrometry and secondary mass spectrometry, with mass acquisition ranges of 100-1000 Da and 50-1000 Da, respectively. A 10 mmol/L sodium formate solution was used as the mass correction solution in both the positive and negative ion modes. Library View software was used to establish the precursor ion accurate quality database and the product ion fragment mass spectrometry database of the corresponding characteristic components of the different kinds of TCM. In the Library View database software, the name of each characteristic component of the 62 kinds of TCM was input (serial number) in order to classify the screened characteristic components. The samples were processed using the same method and analyzed. Peak View software was used to rapidly analyze and screen the target components of the TCM. The high-resolution data collected from the samples to be tested were imported into the Peak View software, followed by the compound list of the established MS database of standard medicinal materials. After setting the identification method parameters and library retrieval parameters, a matching analysis was performed, and the candidate substances for each peak were automatically identified by comparing the mass spectrum, accurate molecular ion mass number, fragment ion mass number, retention time, and other related parameters. The determination conditions of compound detection were as follows: the comprehensive score was more than 70 points. The molecular formula, retention time, mass spectrum as well as the primary isotope mass spectrometry, primary mass spectrometry, and secondary mass spectrometry data were matched with the library compounds. The corresponding list of “TCM-characteristic components” was established, and a high-resolution MS library of 388 characteristic components from the 62 types of TCM was constructed. Each TCM contains 5-10 characteristic components. According to the screening analysis of the actual food samples of the prepared wine, substitute tea, and beverage, one batch of the prepared wine sample matched with seven characteristic components of epimedium, and it was inferred that epimedium was added to the prepared wine samples. This method can allow for the qualitative screening of TCM without standards and has the characteristics of high throughput, accuracy, simplicity, and rapidity. It solves the difficulty in identifying and confirming illegally added TCM in food; provides technical methods and a basis for cracking down on the illegal addition of TCM in food; and facilitates the rapid screening and identification of illegally added TCM in food.

在食品中违法添加中药材对人体健康具有潜在或显性的危害,就中药材的性质而言,中药材都有功能主治、适用人群、用法用量,具有与食品完全不同的功效和服用禁忌。但是,一些食品生产企业受利益的驱动,只顾中药材功效而忽视其毒性和副作用,在所谓的“食疗”、“养生”等食品中违法添加中药材,例如:含有生地的炖鸭煲调料包、含有木香、白术的茶叶、含有女贞子和淫羊藿的椰园海宝(干制水产品)、含有红豆杉和金樱子的配制酒、含有杜仲和淫羊藿的饮料等。

在打击向普通食品和保健食品中违法添加中药材的行为过程中,监管部门执法人员只能通过查看产品配料表和到企业现场巡查等最传统的方式来判断食品中是否违法添加中药材,但往往会因生产企业不如实标注配料表或生产现场对非法添加的中药材原料进行隐藏等情况,形成监管盲区。因此,食品中非法添加中药材的鉴别工作在技术手段上面临巨大的挑战。

目前中药材鉴别手段,主要有性状鉴别^[1]^、显微鉴别^[2,3,4]^、薄层鉴别^[5,6,7]^、中药材特征图谱^[8,9,10,11,12,13]^或指纹图谱鉴定^[14,15,16,17]^、DNA条形码分子鉴定^[18,19,20,21]^等方法,但是这些手段都是对中药材原料的真伪鉴别,而对食品中违法添加中药材的识别和鉴别,从本质上可追溯为鉴定其违法添加中药材的化学成分,而由于食品原料的多样性、食品基质和加工工艺的复杂性、加入的中药材品种未知性等原因,导致所添加的中药材在食品中可能与中药材原料中的特征图谱或指纹图谱发生了变化,因而其不适用于食品中违法添加中药材的鉴别。而目前在中药材应用较广的DNA条形码分子鉴定,是利用基因组中一段公认的、相对较短的DNA序列来进行物种鉴定,但其在中药材的鉴定中也具有一定的挑战,中药材采收部位和时期的不同,导致DNA在组织中的降解程度不同,储存时间和贮藏养护方式不当也会影响中药材质量,从而影响DNA条形码序列,而食品因其基质不同以及加工工艺可能存在高温和干燥过程的影响,均可能对中药材的DNA条形码序列造成影响,因此,单纯凭借DNA条形码分子鉴定食品中的中药材具有一定的局限性。

随着质谱技术的发展,高效液相色谱-四极杆飞行时间质谱(HPLC-Q-TOF/MS)具有分析速度快、灵敏度和分辨率高、多组分同时测定的优点,在分离分析复杂食品基质中的内源性组分和外来添加物中具有显著优势,大大提升了色谱-质谱联用技术的适用性,甚至不需要对照品,即可通过高精度的谱图信息解析产品的特征组分。虽然,现在也存在中药材中部分组分的质谱库,但其所含化合物数量少,且化合物未与中药材进行关联,难以实现对食品中中药材的快速鉴别。

本研究通过建立“中药材-特征组分”对应清单,构建了食品中常非法添加的62种中药材中388种特征组分质谱库,开发出一种基于HPLC-Q-TOF/MS信息的食品中中药材鉴别方法,解决了食品中非法添加中药材难以识别和确证的难题。

## 1 实验部分

### 1.1 仪器、试剂与材料

液相色谱(Dionex Ultimate 3000)-四极杆-飞行时间质谱仪(SCIEX Triple TOF 5600+),配备Analyst 1.6工作站、Peak View定性筛查软件、Library View数据库软件(美国SCIEX公司); Allegra X-15R型离心机(美国Beckman公司); EDAA-2600T型超声波清洗器(上海安谱科学仪器有限公司);涡旋混合器(美国TALBOYS公司); ME2002E分析天平(梅特勒-托利多国际贸易上海有限公司)。

62种对照药材:土茯苓、大蓟、女贞子、山茱萸、川牛膝、川芎、丹参、五味子、升麻、巴戟天、木香、车前草、平贝母、玄参、生地黄、白及、白术、白芍、地骨皮、红花、吴茱萸、杜仲、沙苑子、牡丹皮、苍术、补骨脂、诃子、远志、佩兰、刺五加、泽泻、知母、罗布麻、金荞麦、金樱子、厚朴、枳壳、枳实、茜草、首乌藤、骨碎补、桑白皮、益母草、淫羊藿、菟丝子、银杏叶、黄芪、湖北贝母、番泻叶、蒲黄、蒺藜、墨旱莲、刺玫果购自中国食品药品检定研究院,侧柏叶购自成都普菲德生物技术有限公司,熟大黄、麦冬、天冬、购自成都德思特生物技术有限公司,芦荟、红景天、积雪草、柏子仁、桑枝购自中国药品生物制品检定所,62种中药材样品和配制酒、代用茶、饮料样品购自电商平台,0.22 μm有机系滤膜(天津津腾公司),乙腈、甲醇均为色谱纯(德国Merck公司),甲酸为质谱纯(美国Fisher Scientific公司),超纯水(电阻率为18.2 MΩ·cm, 25 ℃)(美国Millipore公司);其他试剂均为分析纯。

### 1.2 仪器条件

1.2.1 高效液相色谱条件

色谱柱:Thermo Accucore aQ色谱柱(150 mm×2.1 mm, 2.6 μm);柱温:35 ℃;进样量:5 μL;流速:0.3 mL/min;正离子扫描(ESI^+^)模式:流动相A为乙腈,流动相B为0.1%甲酸水溶液;负离子扫描(ESI^-^)模式:流动相A为乙腈,流动相B为超纯水;采用二元梯度洗脱,洗脱程序:0~2 min, 5%A; 2~20 min, 5%A~95%A; 20~25 min, 95%A; 25~26 min, 95%A~5%A; 26~30 min, 5%A。

1.2.2 TOF/MS工作条件

离子源:电喷雾离子源;ESI^+^模式下喷雾电压5500 V;ESI^-^模式下喷雾电压4500 V;离子源温度550 ℃;气帘气241 kPa (35 psi);雾化气:379 kPa(55 psi);辅助气:379 kPa(55 psi);去簇电压:60 V;碰撞能量:35 V;扫描方式采用全扫描一级质谱,质量采集范围100~1000 Da;全扫描二级质谱,质量采集范围50~1000 Da。质量数校正液为10 mmol/L甲酸钠溶液。

### 1.3 62种“中药材-特征组分”对应清单的构建

依据卫生部关于进一步规范保健食品原料管理的通知(卫法监发[2002]51号)^[22]^中可用于保健食品的物品名单,确定食品中可能非法添加的62种中药材原料清单,通过查阅文献确定各中药材多种特征组分对应清单,每种中药材包括5~10种特征组分,形成“中药材-特征组分”对应清单。

### 1.4 62种中药材特征组分质谱数据库的构建

1.4.1 对照药材提取液的制备

分别称取62种对照药材1.0 g于50 mL离心管中,加入10 mL甲醇超声提取10 min,静置30 min后于4000 r/min离心5 min,取上清液过0.22 μm有机系滤膜。

1.4.2 中药材特征组分高分辨质谱库的建立

依据“中药材-特征组分”对应清单,将对照药材特征组分质谱图加入液相色谱-质谱数据库中:于Library View数据库软件中输入每种对照药材各特征组分的名称(采用对照药材名称(序号)-特征组分名称的命名方式以期对筛查出的特征组分进行药材归属,如:淫羊藿1-淫羊藿苷、淫羊藿2-朝藿定A、淫羊藿3-朝藿定B、淫羊藿4-朝藿定C、淫羊藿5-宝藿苷Ⅰ、淫羊藿6-宝藿苷Ⅱ、淫羊藿7-2″-*O*-鼠李糖基淫羊藿次苷Ⅱ)、英文名称、CAS号、分子式、精确相对分子质量,建立对照药材各特征组分的一级精确质量数据库;将采集的二级碎片离子图谱添加至Library View数据库软件中相应的特征组分化合物项下,由此建立对照药材各特征组分的二级碎片离子谱库,包括离子采集模式、碰撞能量和特征碎片离子信息。

### 1.5 样品前处理与靶向筛查分析

1.5.1 样品前处理

称取1.0 g样品于50 mL离心管中,加入10 mL甲醇超声提取10 min,静置30 min后于4000 r/min离心5 min,取上清液过0.22 μm有机系滤膜。

1.5.2 样品靶向筛查分析

将待测样品采集到的高分辨数据导入Peak View软件,将建立好的对照药材特征组分质谱数据库化合物列表导入到Peak View软件,设置鉴别方法参数、库检索参数后进行匹配分析:化合物提取离子流响应强度>100或信噪比(*S/N*)>5,母离子精确相对分子质量和二级碎片质量数偏差设为±10 ppm,保留时间偏差设为±2.5%;数据库综合得分设置:质量数偏差占比为20%,保留时间偏差占比为20%,同位素比值占比为20%,分子式匹配占比为20%,数据库匹配占比为20%。通过与数据库中的MS/MS谱图及精确分子离子质量数、碎片离子质量数、保留时间等相关参数对比而自动鉴定每个峰的候选物质。化合物检出判定条件:综合得分>70分,经人工检查化合物分子式、保留时间、一级同位素峰、一级精确质量数和二级碎片与谱库化合物全部匹配。

## 2 结果与讨论

### 2.1 色谱流动相的优化

ESI^+^模式下,比较考察了乙腈-5 mmol/L乙酸铵和乙腈-0.1%甲酸两种流动相体系,部分化合物在乙腈-5 mmol/L乙酸铵流动相体系下峰形不佳出现拖尾现象,且峰响应较差,故ESI^+^模式下选择乙腈-0.1%甲酸流动相体系。

ESI^-^模式下,比较考察了乙腈-水和乙腈-0.1%氨水两种流动相体系,部分化合物在乙腈-0.1%氨水流动相体系下峰形不佳峰宽较大,响应受到抑制,故ESI^-^模式下选择乙腈-水流动相体系。

### 2.2 “中药材-特征组分”对应清单表的建立

依据监管信息、新闻报道、文献资料,并结合前期研究初步形成可能非法添加的白术、杜仲、淫羊藿等中药材原料及其多种特征组分对应清单,并购买原料进行验证。最终形成的“中药材-特征组分”对应清单见表1。

**表 1:** “中药材-特征组分”对应清单表

No.	Traditional Chinese medicine (TCM)		Characteristic components
1	Smilacis Glabrae Rhizoma (土茯苓)		astilbin, isoastilbin, taxifolin, resveratrol, isoengelitin
2	Cirsii Japonici Herba (大蓟)		buddleoside, tracheloside, tilianin, pectolinarin, pectolinarigenin
3	Ligustr Lucidi Fructus (女贞子)		ligustroflavone, neonuezhenide, specnuezhenide, nuezhenidic acid, verbascoside, cimidahurinine
4	Corni Fructus (山茱萸)		loganin, daucosterol, cornuside, verbenalin, sweroside, loniceroside
5	Cyathulae Radix (川牛膝)		cyasterone, daucosterol, podecdysone B, ferulic acid, scoparone
6	Chuanxiong Rhizoma (川芎)		ligustrazin, senkyunolide H, levistilide A, ligustilide, senkyunolide I
7	Salviae Miltiorrhizae Radix et Rhizoma (丹参)		tanshinone ⅡA, tanshinone Ⅰ, salvianolic acid A, dihydrotanshinone Ⅰ, salvianolic acid B, cryptotanshinone, tanshinone ⅡB
8	Schisandrae Chinensis Fructus (五味子)		schisandrin C, schisandrin B, schisandrin A, schisanhenol, schizandrol B, schisandrin, anwulignan
9	Cimicifugae Rhizoma (升麻)		cimicifugc acid, ferulic acid, 27-deoxyactein, cimifugin, β-sitosterol, daucosterol
10	Morindae Officinalis Radix (巴戟天)		monotropein, desacetyl asperulosidic acid, asperuloside, asperulosidic acid, piperidine-2-carbonitrile hemioxalate, physcion
11	Aucklandiae Radix (木香)		costol, alantolactone, isoalantolactone, dehydrocostus lactone, costunolide, daucosterol
12	Plantaginis Herba (车前草)		homoplantaginin, plantamajoside, asperuloside, luteolin, luteoloside, cosmosiin
13	Fritillariae Ussuriensis Bulbus (平贝母)		peimine, peimisine, pingbeimine C, edpetiline, pingbeimine A
14	Scrophulariae Radix (玄参)		harpagosid, cinnamic acid, verbascoside, aucubin, β-sitosterol
15	Rehmanniae Recens Radix (生地黄)		verbascoside, jionoside B1, isoacteoside, isoacteoside, daucosterol
16	Bletillae Rhizoma (白及)		blestriarene A, blestriarene B, schizandrin, physcion, daucosterol, syringaresinol
17	Atractylodis Macrocephalae Rhizoma (白术)		atractylenolide Ⅰ, atractylenolide Ⅱ, atractylenolide Ⅲ, atractylenolide Ⅳ, atractyline, β-sitosterol, taraxeryl acetate
18	Paeoniae Radix Alba (白芍)		paeoniflorin, benzoylpaeoniflorin, galloylpaeoniflorin, daucosterol, albiflorin, oxypaeoniflora, lactiflorin
19	Lycii Cortex (地骨皮)		scopoletin, kukoamine A, kukoamine B, scopoletin, ferulic acid
20	Carthami Flos (红花)		safflor yellow A, hydroxysafflor yellow A, apigenin, daucosterol, carthamone, β-sitosterol
21	Rhodiolae Crenulatae Radix et Rhizoma (红景天)		salidroside, rhodionin, picein, arbutin, kaempferol, isoquercitrin, ellagic acid
22	Evodiae Fructus (吴茱萸)		rutaecarpine, evodiamine, limonin, dehydroevodiamine, dihydroevocarpine, evocarpine, hydroxyevodiamine, evodine, limonin
23	Eucommiae Cortex (杜仲)		geniposidic acid, chlorogenic acid, caffeic acid, eucommiol, pinoresinol diglucoside
24	Astragali Complanati Semen (沙苑子)		complanatoside, 4'-methoxyresveratrol, calycosin, formononetin, complanatoside A, rhamnocitrin, myricomplanoside, deoxyrhapontin
25	Moutan Cortex (牡丹皮)		gallic acid, paeoniflorin, benzoylpaeoniflorin, kaempferide, isorhamnetin, paeonol
26	Aloe (芦荟)		aloe emodin, chrysophanic acid, aloesin, aloin A, emodin, rhein, aloenin
27	Atractylodis Rhizoma (苍术)		atractyline, atractylodin, atractylenotide Ⅲ, atractylodinol, acetyl-atractylodinol
28	Psoraleae Fructus (补骨脂)		psoralen, bavachalcone, corylin, bavachromene, bavachinin A, psoralidin, bakuchiol, bavachromanol
29	Chebulae Fructus (诃子)		arjunolic acid, arjungenin, chebulinic acid, ethyl gallate, gallic acid, chebulagicacid
30	Polygalae Radix (远志)		senegenin, tenuifoliside A, tenuifoliside B, polygalacic acid, tenuifolin, sibiricose A5, sibiricose A6, polygalaxanthone Ⅲ, 7-O-methylmangiferin, 1,2,3,7-tetramethoxyxanthone
31	Ophiopogonis Radix (麦冬)		ophiopogonin D', ophiopogonanone C, ophiopogonanone E, methylophiopogonanone, methylophiopogonone A
32	Eupatorii Herba (佩兰)		p-cymene, neryl acetate, mannitol, 2-hydroxycinnamic acid, heliotrine, thymol
33	Acanthopanacis Senticosi Radix et Caulis (刺五加)		eleutheroside E, isofraxidin, eleutheroside B1, eleutheroside C, daucosterol
34	Alismatis Rhizoma (泽泻)		alisol B 23-acetate, alisol A 24-acetate, alisol C 23-acetate, alisol A, alisol B
35	Anemarrhenae Rhizoma (知母)		timosaponin B-Ⅱ, sarsasapogenin, mangiferin, aurantiamide, anemarrhenasaponin A2, timosaponin A-Ⅲ, anemarsaponin E
36	Apocynum venetum (罗布麻)		quercetin-3-O-sophoroside, kaempferol, quercetin, isoquercitrin, rutin
37	Fagopyri Dibotryis Rhizoma (金荞麦)		alnusone, procyanidin B2, L-epicatechin, catechin, protocatechuate
38	Rosae Laevigatae Fructus (金樱子)	hyperoside, hesperidin, daucosterol, liquiritigenin, luteolin, quercetin, rutin, ursolic acid, protocatechuate, aurin	
39	Magnoliae Officinalis Cortex (厚朴)	magnolol, honokiol, magnolignan, magnocurarine, syringin	
40	Aurantii Fructus (枳壳)	neohesperidin, naringin, hesperidin, narirutin, hordenine, 7-hydroxycoumarin, meranzin, rutoside	
41	Aurantii Fructus Immaturus (枳实)	naringin, hesperidin, neohesperidin, narirutin, N-methyltyramine, 5,7-dihydroxycoumarin, rhoifolin, scopoletin	
42	Platycladi Semen (柏子仁)	pinusolide, cianidanol, daucosterol, β-sitosterol, quercetin	
43	Rubiae Radix (茜草)	mollugin, purpurin, dihydromollugin, alizarin, 2-methylanthraquinone, oleanic acid, physcion	
44	Polygoni Multiflori Caulis (首乌藤)	2,3,5,4'-tetrahydroxy stilbene 2-O-glucoside, polydatin, resveratrol, emodin, physcion, aloe emodin, rhein, emodin-8-glucoside, L-epicatechin	
45	Drynariae Rhizoma (骨碎补)	naringin, eriodictyol, neoeriocitrin, astragalin, afzelin	
46	Mori Cortex (桑白皮)	sanggenon C, mulberroside A, oxyresveratro, kuwanon G, sanggenone H	
47	Ramulus Mori (桑枝)	cyclomulberrin, dibydromorin, mulberroside A, cyclomulberrochromene, 1-deoxynojirimycin, morusin, oxyresveratrol, kuwanon G	
48	Leonuri Herba (益母草)	stachydrine, leonurine, ajugoside, xanthotoxin, isopimpinellin, rutoside, quercetin, isoquercitrin	
49	Centellae Herba (积雪草)	asiatic acid, madecassic acid, asiaticoside, madecassoside, asiaticoside B, kaempferol	
50	Epimedii Folium (淫羊藿)	icariin, epimedin A, epimedin B, epimedin C, baohuoside Ⅰ, baohuoside Ⅱ, 2″-O-rhamnosyl icariside Ⅱ	
51	Cuscutae Semen (菟丝子)	hyperoside, 4-dicaffeoylquinic acid, isorhamnetin, astragalin, quercetin	
52	Ginkgo Folium (银杏叶)	ginkgolide A, ginkgolide B, ginkgolide C, ginkgolic acid, bilobalide	
53	Astragali Radix (黄芪)	methylnissolin, licoagroside D, isomucronulatol, calycosin-7-glucoside, pratensein 7-O-glucopyranoside, astraisoflavan-7-O-β-D-glucoside, astragaloside IV, astragaloside I, astragaloside Ⅱ, astragaloside Ⅲ	
54	Fritillariae Hepehensis Bulbus (湖北贝母)	peimine, peiminine, hupehenine, zhebeirine, sipeimine-3β-D-glucoside, coelogin	
55	Sennae Folium (番泻叶)	sennoside A, sennoside B, sennoside C, aloe emodin, rhein	
56	Typhae Pollen (蒲黄)	typhaneoside, isorhamnetin-3-O-neohespeidoside, isorhamnetin, isoprhamnetin-3-rutinoside, naringenin, quercetin	
57	Tribuli Fructus (蒺藜)	harmine, hecogenin, tiliroside, tigogenin, gitogenin	
58	Ecliptae Herba (墨旱莲)	ecliptasaponin A, demethylwedelolactone, wedelolactone, luteoloside, luteolin, acacetin	
59	Rhei Radix Et Rhizoma (熟大黄)	aloe emodin, rhein, emodin, chrysophanol, physcion, gallic acid	
60	Asparagus Rad (天冬)	sarsasapogenin, diosgenin, gracilline, daucosterol, β-sitosterol, stigmasterol	
61	Dahurian Rose Fruit (刺玫果)	hyperoside, β-sitosterol, oleanic acid, uosolic acid, rutin	
62	Platycladi Cacumen (侧柏叶)	hinokiflavone, α-cuparenol, α-biotol, quercetin, neocryptomerin, quercitrin	

### 2.3 中药材特征组分高分辨质谱数据库的建立

自建质谱库中每种对照药材包括5~10种特征组分的一级精确质量数据库和二级碎片离子谱库,共计62种中药材中388种特征组分。部分信息见表2,具体信息见附表(http://www.chrom-China.com)。

**表2:** 62种中药材特征组分信息表^*^

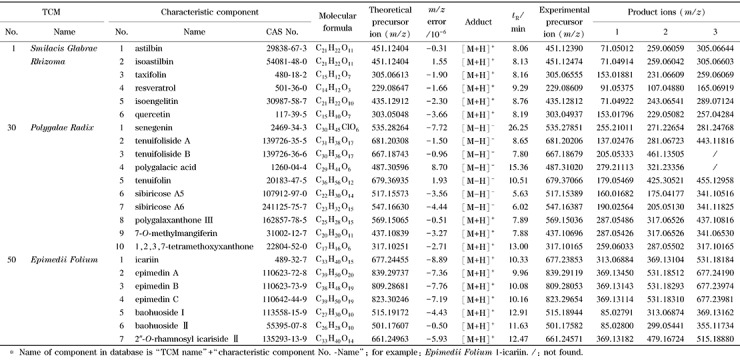

### 2.4 样品靶向筛查分析

采用本文建立的方法对市售62种中药材样品进行分析筛查验证,62种中药材样品高分辨数据均可分别与已建立的62种对照药材特征组分匹配一致,说明所建立的方法可有效筛查鉴别中药材。

根据监管信息、新闻报道并结合前期研究基础,常非法添加中药材的食品主要有配制酒、代用茶以及饮料,故对市售的10批次配制酒样品、10批次代用茶样品和10批次饮料样品进行分析筛查,其中1批次配制酒样品筛查匹配的化合物与淫羊藿药材7种特征组分质谱信息匹配一致,推断该配制酒样品中可能加入淫羊藿中药材。该配制酒样品靶向筛查结果见表3,配制酒样品中淫羊藿7种特征组分与淫羊藿对照药材7种特征组分碎片离子镜像图见图1((上)样品中淫羊藿特征组分碎片离子图,(下)对照药材中相应的淫羊藿特征组分碎片离子图)。

**表3:** 配制酒样品与对照药材特征成分高分辨质谱数据库靶向筛查分析结果表

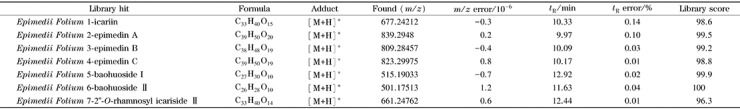

**图 1:**
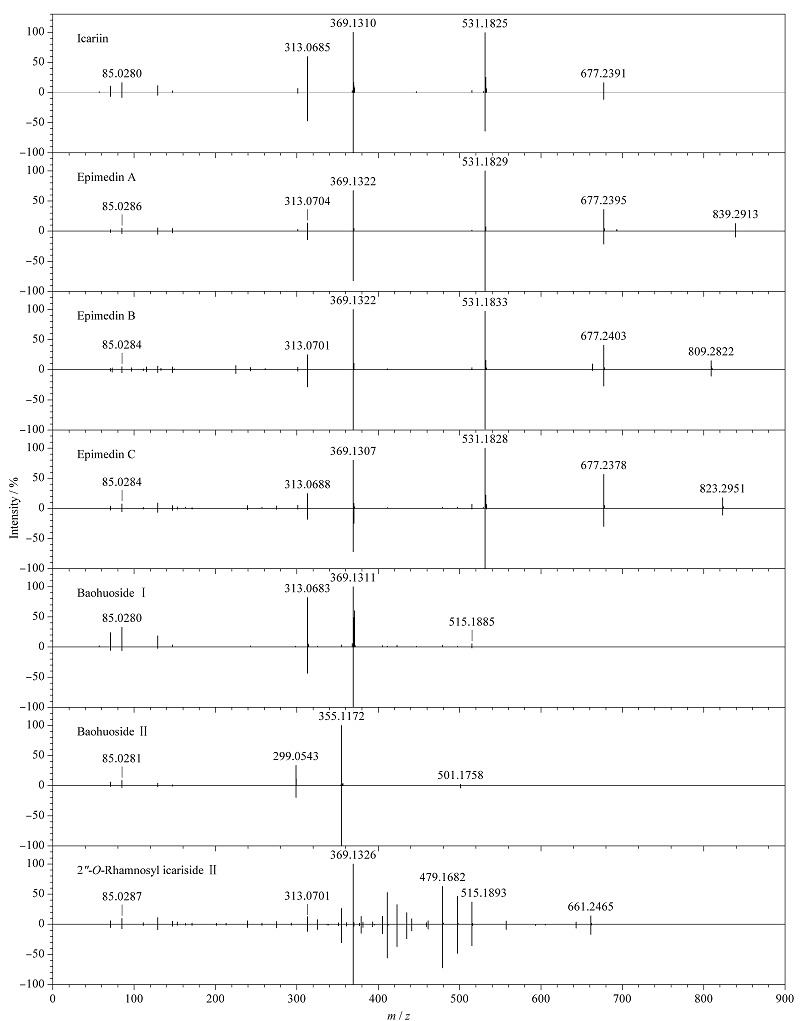
配制酒样品中淫羊藿特征组分的碎片离子镜像图

## 3 结论

本文开发出一种高效液相色谱-四极杆-飞行时间质谱仪快速筛查鉴别食品中非法添加的62种中药材的方法,本方法可以实现无标准品情况下中药材的定性筛查,具有高通量、准确、简便、快捷等特点;针对监管实际中常违法添加的中药材原料进行重点研究,解决了食品中非法添加的中药材难以识别和确证的难题,以及原有利用个别标志物无法初步反推添加的原料品种问题,克服了监管部门执法人员只能通过查看产品配料表和到企业现场巡查等最传统的方式来判断食品中是否违法添加中药材造成的监管盲区,为打击食品中非法添加中药材违法行为提供技术方法和依据。

研究中所选定的易非法添加对照药材种类目前仅包括可用于保健食品不可用于食品的种类,在后续的研究过程中应该进一步增加中药材种类,包括不可用于保健食品的中药材,更全面地扼制食品中违法添加中药材行为的发生或发展,为监管提供更全面的技术支持。
